# Telehealth pulmonary rehabilitation: A review of the literature and an example of a nationwide initiative to improve the accessibility of pulmonary rehabilitation

**DOI:** 10.1177/1479972317724570

**Published:** 2017-08-08

**Authors:** A-M Selzler, J Wald, M Sedeno, T Jourdain, T Janaudis-Ferreira, R Goldstein, J Bourbeau, MK Stickland

**Affiliations:** 1 Department of Medicine, Faculty of Medicine, University of Alberta, Edmonton, Canada; 2 Faculty of Physical Education and Recreation, University of Alberta, Edmonton, Canada; 3 Montreal Chest Institute, McGill University Health Centre (MUHC), Montreal, Canada; 4 Respiratory Epidemiology and Clinical Research Unit (RECRU), Montreal, Canada; 5 G.F. MacDonald Centre for Lung Health, Edmonton, Canada; 6 School of Occupational Therapy, McGill University, Montreal, Canada; 7 Translational Research in Respiratory Diseases Program, Research Institute of the McGill University Health Centre, Montreal, Canada; 8 Department of Medicine, University of Toronto, Canada; 9 West Park Healthcare Centre, Toronto, Canada

**Keywords:** Pulmonary rehabilitation, chronic obstructive pulmonary disease, telehealth, chronic disease management, accessibility

## Abstract

Several different applications of telehealth technologies have been used in the care of respiratory patients, including telemonitoring, teleconsultations, tele-education, and telehealth-pulmonary rehabilitation (PR). Telehealth technology provides an opportunity to assist in the management of chronic respiratory diseases and improve access to PR programs. While there is inconclusive evidence as to the effectiveness of telemonitoring to reduce healthcare utilization and detection of exacerbations, teleconsultations have been shown to be an effective means to assess patients’ disease prior to the initiation of PR, and telehealth PR has been shown to be as effective as institution-based PR at improving functional exercise capacity and health-related quality of life. To improve PR access across Canada and ensure a high standard of program quality, a team of clinicians and researchers has developed and begun to implement a national standardized PR program that can be delivered across different settings of practice, including remote satellite sites via telehealth PR. The program has adapted the “Living Well with COPD” self-management program and includes standardized reference guides and resources for patients and practitioners. A progressive and iterative process will evaluate the success of program implementation and outcomes. This initiative will address nationwide accessibility challenges and provide PR content as well as evaluations that are in accordance with clinical standards and established self-management practices.

## Introduction

Pulmonary rehabilitation (PR) is an essential component of the long-term management of chronic obstructive pulmonary disease (COPD).^1^ The benefits of PR are substantial, including reduced patient symptoms, as well as improved patient exercise capacity, health status, knowledge, and self-efficacy.^2–7^ PR has also been shown to reduce healthcare costs and hospital admissions due to COPD exacerbations.^8–11^ However, many countries have documented widespread underutilization of PR services.^12^ In addition to poor commissioning of PR programs and lack of patient and practitioner knowledge of the availability and benefits of PR, one repeatedly identified reason for this gap is poor program accessibility.^13–15^

Current guidelines recommend PR for all patients with COPD who experience ongoing symptoms despite optimal pharmacologic therapy, and PR is increasingly recommended for a wide range of non-COPD chronic respiratory conditions.^7^ However, the capacity of PR programs is dramatically lower than the estimated number of people with respiratory diseases who could benefit from participating in PR. In Canada, it is estimated that the national capacity of PR programs is 10,280 people per year, which translates into only 0.4% of the estimated population of patients with COPD having access to PR.^16^ A systematic review^12^ of the national program capacity across four countries—the United Kingdom, Canada, New Zealand, and Sweden—reported that ≤1.2% of the estimated population of COPD has access to PR. Given the clear benefits of PR and the increasing impact of COPD on morbidity and mortality,^17,18^ strategies to improve the access and capacity of PR are needed.

As PR programs tend to be located in urban areas,^16^ people living in rural and remote centers have limited access to PR. This is a particularly pressing issue in countries such as Canada where there is a large geographical area with low population density and few health centers in rural areas. Several advances in the delivery of PR have been implemented to improve access to such programs, including PR delivered with telehealth technology. Telehealth technologies allow for distribution of healthcare services and exchange of information between a healthcare provider and a patient in different geographical locations and provide an important avenue to reach people who live in rural communities.^19^ Several different applications of telehealth technology have been used to provide health services to people with chronic respiratory diseases; such applications include telemonitoring, teleconsultation, tele-education, and telehealth PR. Each application has its own unique purpose, procedure, benefits, challenges, and evidence of effectiveness. This article reviews the application of telehealth technology to deliver healthcare services to chronic respiratory patients and describes how a team of clinician-researchers propose to address accessibility challenges through telehealth technology, while ensuring program quality and evaluating the effectiveness of a new PR program.

## Applications of telehealth technologies

Telemonitoring utilizes equipment, sensors, and surveys within patient’s homes, which are subsequently transmitted in real time or in a retrospective manner to a healthcare professional (HCP) or team at a central location.^20,21^ Information is then monitored and evaluated by the HCP and patients are informed if there is evidence to suggest worsening of their condition. It has been proposed that telemonitoring may be particularly useful in the management of COPD as it may allow for early detection of exacerbations and subsequently a decrease in healthcare utilization.^22^ Both COPD patients and HCP tend to have positive experiences with telemonitoring. Patients report feeling satisfied with the process^23,24^ and the ease of equipment use;^25,26^ they also report feeling empowered to be intimately involved in the self-management of their disease.^27^ While some studies have reported that telemonitoring lead to positive outcomes, including reduced exacerbations^28^ and hospitalizations,^29–31^ the effectiveness of telemonitoring is not universally established.^28,32,33^ Rather than supporting self-management of one’s disease, telemonitoring may foster dependence on HCP to interpret symptoms and manage one’s disease.^33^ Future studies that are well designed, with long-term follow-up and large sample sizes, are needed to determine the effectiveness of telemonitoring on health outcomes in chronic respiratory patients.^33^

Teleconsultation is used to assess patients’ clinical status and requires videoconferencing software to connect physicians with COPD patients at satellite sites in real time. Teleconsultations often precede telehealth PR to assess patients’ suitability and safety for PR. HCPs at satellite sites may assist with the communication and transmission of clinical information, including chest auscultations, vital signs, pulse oximetry, and tests of functional capabilities. Teleconsultations have been found to be feasible,^34^ reliable,^19,34^ and dramatically reduce the amount of travel by patients.^19^ Teleconsultations are a promising means to connect rural patients with respiratory specialists and provide an exciting opportunity to improve access to consultation services.

Tele-education utilizes web-based platforms to deliver information and services that pertain to the management of patients’ condition. Such platforms can be designed for HCPs to enhance knowledge of disease management program components as well as facilitate the delivery of an intervention to patients. Tele-education platforms can also be designed for patients to facilitate disease self-management knowledge and support the uptake of self-management interventions. There is a growing interest among COPD patients to use web-based and alternative technologies to receive disease-management information. A survey conducted by Rogers et al.^35^ in 75 COPD patients indicated that 77% of respondents wanted to received health and disease-management information using either web or phone-based applications, with 51% preferring to access information with a computer. Given the interest among patients to access information about disease management, it is important that qualified clinicians and researchers develop accurate, useful, and user-friendly resources for patients to access. Currently, there are numerous online education platforms for patients with COPD, including those developed by professional associations, as well as those developed public–private partnerships, such as Living Well with COPD (LWWCOPD; available at LivingWellWithCOPD.com).

Telehealth PR is a type of telehealth intervention that involves the delivery of PR content through various communication modalities (e.g. videoconferencing, telephone), which connects patients to HCPs. Telehealth PR sessions may be delivered within a healthcare institution, or to within the patients’ homes. Telemonitoring may be used in conjunction with telehealth PR; however, the impact of monitoring physiological variables on PR outcomes and patient self-management is unclear. Stickland et al.^34^ compared PR delivered via telehealth to 147 COPD patients with PR delivered in person at a healthcare institution to 262 COPD patients. Teleconsultation was used in the initial patient assessment process, with an HCP at the satellite site performing and communicating chest auscultations, vital signs, pulse oximetry, and tests of functional capabilities to the physician at the host institution via videoconferencing software. The telehealth sites joined the education sessions at the host institution remotely via videoconferencing software and patients received supervised exercising training at a local institution. Both groups demonstrated similar attendance, improvements in exercise capacity, and health-related quality of life (HRQOL). This center-based telehealth PR study provides a useful model to develop future telehealth PR initiatives and may be an important step toward addressing PR accessibility challenges.

There have been several other creative initiatives designed to improve access to PR services, including the delivery of telehealth home-based interventions. Holland et al.^26^ examined the effectiveness of a home-based PR program in patients with moderate COPD. Readily available telehealth equipment was installed in patients’ homes to allow for videoconferencing, along with an exercise bicycle and pulse oximeter. During the in-home exercise sessions, patients monitored their symptoms and a HCP monitored the patient, their heart rate and SpO_2_ via videoconferencing. All participants who completed the 8-week program had clinically significant improvements in exercise capacity and the dyspnea subscale of the Chronic Respiratory Questionnaire. Tsai et al.^36^ conducted a randomized controlled trial of 37 COPD patients that compared an in-home telehealth PR program to a no PR control group. This intervention included telemonitoring, telehealth PR, and telehealth consultations. The telehealth PR program included in-home virtual supervision of walking, cycling on an exercise bike, and strength training. The telehealth PR group had improved exercise capacity, self-efficacy, and mood at the end of the intervention, as compared to the non-PR control group. However, clinically and statistically significant improvements in HRQOL were not observed, which may indicate that some aspect of the intervention environment does not mimic that of traditional institution-based PR. While not PR, Nield and Hoo^37^ conducted a randomized controlled trial to determine the effectiveness of a pursed-lip breathing intervention delivered via Skype™ software. The 4-week intervention included feedback on the technique as well as instruction on the application of pursed-lip breathing to activities of daily living, such as walking. The intervention group had a decrease in dyspnea intensity and increase in social support; however, no differences in functional exercise capacity, dyspnea severity, or dyspnea distress were observed between the experimental and control groups.

These telehealth intervention and PR studies illustrate the potential of institution-based and in-home virtual telehealth programs to effectively delivery PR to the large number of rural and remote patients who are unable to access a specialized PR institution. There is also the potential of delivering institution-based telehealth PR to other urban health centers, such as primary care medical centers. This approach could expand the delivery of PR services within urban communities and reduce the need for specialized institutions. Further, it would ensure that community-based programs are supporting the delivery of evidence-based, disease-specific information and practices. Clearly, telehealth PR has the potential to be an important vehicle to increase the accessibility of PR services to both rural and urban patients. However, well-designed studies with careful consideration of intervention content are needed to progress our understanding of the delivery and effectiveness of such programs.

## Self-management for behavior modification in pulmonary rehabilitation

The quality of PR content and delivery is fundamental to successfully improve health and behavioral outcomes. The most recent definition of PR put forward by the American Thoracic Society/European Respiratory Society (ATS/ERS) specifies that PR comprises patient tailored therapies “…that include, but are not limited to, exercise training, education, and behavior change, designed to improve the physical and psychological condition of people with chronic respiratory disease and to promote the long-term adherence to health-enhancing behaviors.”^7^ This definition places an emphasis on the importance of behavior change in the long-term management of chronic respiratory diseases. However, the improvements charted throughout PR typically diminish as soon as 6 months after completion,^10,38,39^ and PR maintenance programs have been unsuccessful in sustaining improvements.^40–42^ Furthermore, the impact of PR on health behaviors such as physical activity, breathing management, medication adherence, exacerbation self-management as well as on the motivational predispositions that might drive behavior change remains unclear, despite the latter being considered a key deliverable.

There is evidence to suggest that self-management training in conjunction with PR translates into positive outcomes, including improved quality of life, dyspnea, and healthcare utilization.^43,44^ The purpose of self-management education within PR is to guide patients through the acquisition and implementation of skills that will help them perform the health behaviors required for them to independently manage their disease. These skills often include goal setting, problem-solving, and identifying and acting on collaboratively determined action plans.^7^ LWWCOPD is an evidenced-based program designed to improve patient disease self-management. The program focuses on improving pre-disposing factors (i.e. patient knowledge, beliefs, motivation, self-efficacy) and enabling factors (i.e. skills, accessibility) of behavior change. The LWWCOPD program has been shown to reduce exacerbations, hospitalizations, and emergency department visits, as well as improve HRQOL in patients with COPD.^45–47^

## Nationwide initiative to improve access to PR

To address PR capacity and accessibility challenges^16^ within Canada and to ensure that evidenced-based self-management education is being provided to all patients receiving PR services, a group of clinicians and researchers have come together to develop a standardized PR program that can be used within different settings of practice, including traditional PR centers, primary care medical centers, and satellite sites (i.e. sites that are remote from major institutions) with the use of telehealth and web-based resources. The goal of the initiative is (1) to improve ease of access/clinical implementation of PR, (2) to ensure PR program quality, and (3) to ensure that standard PR program content is in accordance with clinical standards and established self-management practices. The program includes web-based comprehensive resources for patients and HCPs, with reference guides on approaches for (1) pre-program evaluation/physician consultation, (2) exercise training, (3) interactive, patient-centered education to support disease self-management, (4) post-program evaluation, and (5) follow-up and maintenance. Importantly, the program has adapted LWWCOPD for the self-management education component of the PR program, which includes educational handouts based on adult education methods, as well as educational presentations that can be delivered to patient(s) in-person by an HCP or via videoconferencing software. The unique aspect of this program is that the evidenced-based self-management intervention material will be made accessible to support different mediums of delivery, including in-person and web-based platforms.

The Obesity-Related Behavioral Intervention Trials (ORBIT) model^48^ is guiding the development of the new standardized Canadian PR program. The purpose of the ORBIT model is to provide a structured framework for developing behavioral interventions that optimizes and integrates fundamental behavioral and social sciences research into practically relevant treatments to prevent/manage chronic diseases.^48^ The ORBIT model outlines a progressive and iterative process to evaluate the success at each phase based on pre-determined milestones. Achievement of a milestone represents forward movement and sub-optimal results represents a backward movement to further refine the behavioral treatment, which may include changes to the content or delivery of the program.

In this initiative, HCPs have participated in all phases of the iterative development process. A variety of HCPs have created, reviewed, and provided feedback on the content and delivery of education material and the disposition of material on the program website. Self-management education, based on LWWCOPD material, is the cornerstone of the new standardized PR program. Often HCPs do not have training in behavior change and self-management techniques. The goal in this new PR initiative is to provide adequate resources for HCP to effectively deliver the PR intervention. Therefore, all HCP will receive training specific to the self-management tools used and have access to a researcher that specializes in health behavior change research. Further, to support the HCP in the content and delivery of the PR program, the following tools have been developed: standardized learning materials for patients, reference guides that describe how the intervention is to be delivered by the HCP, and reference guides that describe the process for evaluating health and clinical outcomes among patients. These materials will see that patients obtain consistent messaging, style, and methods of delivery should they receive the self-management education alone or attend in PR. A process evaluation of the adequacy of training and resources made available to ensure that HCPs are adequately supported in the delivery of the program.

HCPs will continue to play a vital role in the implementation of this initiative across the country. Consideration of the needs of HCPs and resources available at satellite sites is fundamental to successful implementation of PR programs. HCPs have indicated that resources (staffing, equipment), time demands, and lack of adequate skills and knowledge are barriers to the implementation and sustainability of telehealth PR programs.^15^ Formal surveys as well as informal interactions are being used to obtain feedback from HCP at each individual site, which will then be carefully considered before continuing with implementation at the next site. Each satellite site has a unique set of resources and staff, and the aim is to provide a program that is adaptable so that it can be implemented in all types of settings.

Currently, two pilot trials are being conducted to determine the effectiveness of the new standardized Canadian PR program before nation-wide implementation begins. The primary outcome of the pilot trials is objectively assessed physical activity, which will be assessed before, immediately after, and 6 months after PR. Secondary outcomes include behavioral indicators (i.e. medication adherence and adherence to exacerbation action plan), pre-disposing factors of behavior change (i.e. knowledge, self-efficacy), and clinical outcomes (i.e. exercise capacity, HRQOL, emergency department visits and hospitalizations).

Given the demonstrated effectiveness of PR programs, improving access to PR is one of the most pressing issues within the respiratory community.^7^ To effectively tackle this issue, collaboration among researchers and HCPs will be paramount, along with the development and implementation of creative strategies to help connect patients to PR programs. The initiative presented represents a significant step-forward in the care of respiratory patients by attempting to address nationwide accessibility challenges and providing PR content and evaluations that are in accordance with clinical standards and established self-management practices.
